# Mutations in the SARS-CoV-2 spike receptor binding domain and their delicate balance between ACE2 affinity and antibody evasion

**DOI:** 10.1093/procel/pwae007

**Published:** 2024-03-05

**Authors:** Song Xue, Yuru Han, Fan Wu, Qiao Wang

**Affiliations:** Key Laboratory of Medical Molecular Virology (MOE/NHC/CAMS), Shanghai Institute of Infectious Disease and Biosecurity, Shanghai Frontiers Science Center of Pathogenic Microorganisms and Infection, School of Basic Medical Sciences, Shanghai Medical College, Fudan University, Shanghai 200032, China; Key Laboratory of Medical Molecular Virology (MOE/NHC/CAMS), Shanghai Institute of Infectious Disease and Biosecurity, Shanghai Frontiers Science Center of Pathogenic Microorganisms and Infection, School of Basic Medical Sciences, Shanghai Medical College, Fudan University, Shanghai 200032, China; Key Laboratory of Medical Molecular Virology (MOE/NHC/CAMS), Shanghai Institute of Infectious Disease and Biosecurity, Shanghai Frontiers Science Center of Pathogenic Microorganisms and Infection, School of Basic Medical Sciences, Shanghai Medical College, Fudan University, Shanghai 200032, China; Key Laboratory of Medical Molecular Virology (MOE/NHC/CAMS), Shanghai Institute of Infectious Disease and Biosecurity, Shanghai Frontiers Science Center of Pathogenic Microorganisms and Infection, School of Basic Medical Sciences, Shanghai Medical College, Fudan University, Shanghai 200032, China

**Keywords:** SARS-CoV-2, RBD mutation, antibody evasion, ACE2 affinity, variant of concern, viral evolution

## Abstract

Intensive selection pressure constrains the evolutionary trajectory of SARS-CoV-2 genomes and results in various novel variants with distinct mutation profiles. Point mutations, particularly those within the receptor binding domain (RBD) of SARS-CoV-2 spike (S) protein, lead to the functional alteration in both receptor engagement and monoclonal antibody (mAb) recognition. Here, we review the data of the RBD point mutations possessed by major SARS-CoV-2 variants and discuss their individual effects on ACE2 affinity and immune evasion. Many single amino acid substitutions within RBD epitopes crucial for the antibody evasion capacity may conversely weaken ACE2 binding affinity. However, this weakened effect could be largely compensated by specific epistatic mutations, such as N501Y, thus maintaining the overall ACE2 affinity for the spike protein of all major variants. The predominant direction of SARS-CoV-2 evolution lies neither in promoting ACE2 affinity nor evading mAb neutralization but in maintaining a delicate balance between these two dimensions. Together, this review interprets how RBD mutations efficiently resist antibody neutralization and meanwhile how the affinity between ACE2 and spike protein is maintained, emphasizing the significance of comprehensive assessment of spike mutations.

## Introduction

Severe acute respiratory syndrome coronavirus 2 (SARS-CoV-2), accounting for the devastating COVID-19 pandemic that burst out worldwide at the end of 2019, is a positive-strand RNA virus that belongs to the coronavirus family. As of 12 October 2023, over 771 million confirmed cases with nearly 7 million deaths due to SARS-CoV-2 and its numerous variants have been reported (count from the website of WHO). Despite the 3ʹ to 5ʹ exoribonuclease proofreading activity of its RNA polymerase (Duffy et al., 2008; Moeller et al., 2022), SARS-CoV-2 achieves a high error rate during replication, which offers great potential to gain mutations (Robson et al., 2020). Given the extremely extensive infected population and chronic infection cases reported in immunodeficient individuals, there is sufficient space at both spatial and temporal scales for SARS-CoV-2 to continuously develop various mutations, resulting in a huge reservoir for viral selection and ultimately drastic changes in viral characteristics (McGrath et al., 2022; Qu et al., 2023a; Riddell and Cutino-Moguel, 2023; Telenti et al., 2021; Tian et al., 2022). Particularly, the viral spike (S) protein, responsible for the virus–host interaction, undergoes extensive amino acid substitution under the pressure of strong immune surveillance generated by natural infection, vaccination, or antibody therapy (Cao et al., 2022b; Carabelli et al., 2023; Focosi et al., 2022; Shrestha et al., 2022; Thorne et al., 2022). Myriads of SARS-CoV-2 variants with various S mutation profiles emerged, reflecting their distinct evolutionary trajectories.

Functional and structural studies have revealed that S protein consists of two functional domains, named S1 (amino acid residues 1–686) and S2 (amino acid residues 687–1,273), respectively, both of which are of great significance for viral entry (Ke et al., 2020; Mannar et al., 2022; Walls et al., 2020; Wrapp et al., 2020; Zhang et al., 2021b). Trimeric S protein forms a spike protruding from the virion surface, while its S1 domain is exposed to recognize and bind to the host receptor, angiotensin-converting enzyme 2 (ACE2) (Scialo et al., 2020). The S-ACE2 interaction is through the receptor binding domain (RBD, amino acid residues 306–534) in the S1 domain (Shang et al., 2020). After receptor recognition and viral attachment, several proteolytic cleavage steps occur to remove the S1 domain (Walls et al., 2020). Meanwhile, the S2 domain gets exposed, facilitating the following membrane fusion and the ultimate viral entry (Jackson et al., 2022; Kumar et al., 2022; Meng et al., 2022).

**Figure 1.**
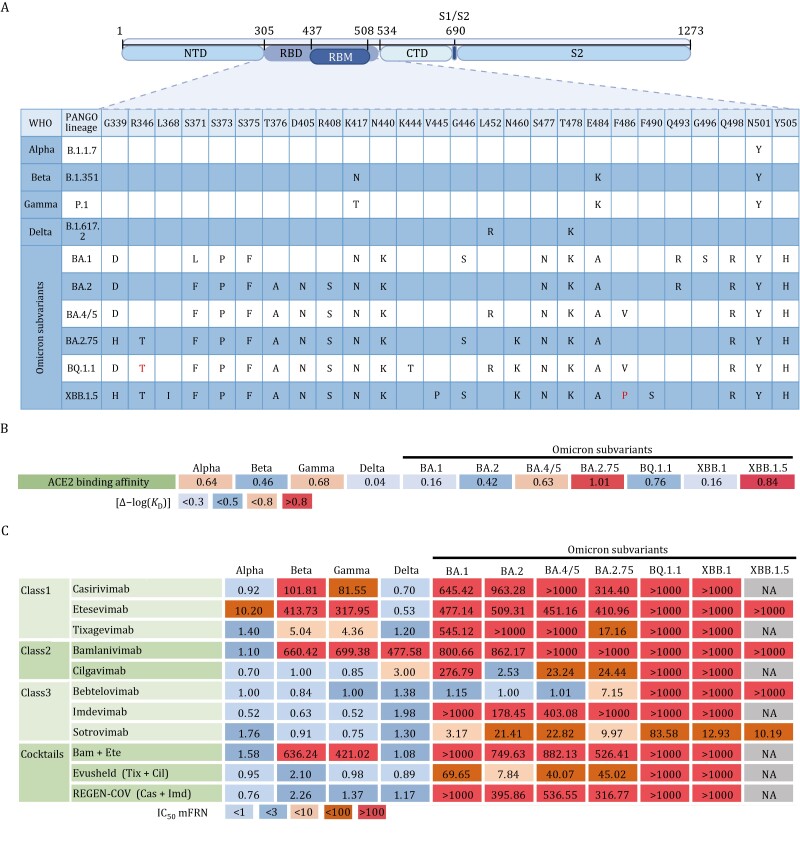
**Various RBD mutations contribute to ACE2 binding and antibody evasion.** (A) The most common mutational profiles for each variant of concern (VOC) within the receptor binding domain (RBD) of SARS-CoV-2 spike (S) protein. Data was obtained from Outbreak.info genomic reports. The amino acid position and the original amino acid of the S protein are shown at the top row of the table. NTD, amino-terminal domain; PANGO, Phylogenetic Assignment of Named Global Outbreak; RBD, receptor- binding domain; RBM, receptor binding motif; S1/S2, junction between the exposed S1 attachment domain and the partially buried S2 fusion domain; WHO, World Health Organization. The R346T and F486P in mutation table indicate the specific mutation of BQ.1.1 and XBB.1.5 relative to their parental strains, BQ.1 and XBB.1, respectively. Created with Biorender. (B) Enhancement of ACE2 affinity for VOCs [Δ−log(*K*_D_) values] with the wildtype as a reference. Means are calculated from published studies reporting ACE2 affinity data of VOCs. Colors represents the level of ACE2 binding enhancement: strong [Δ−log(*K*_D_) > 0.8]; moderate [Δ−log(*K*_D_) = 0.5–0.8]; mild [Δ−log(*K*_D_) = 0.3–0.5]; slightly increased affinity [Δ−log(*K*_D_) < 0.3]. (C) Geometric mean fold reduction in neutralization (mFRN) values of monoclonal antibodies (mAbs) against VOCs relative to the wildtype reference. Means are calculated from published studies reporting neutralization data for the indicated mAbs. Full datasets are available in Supplementary Data File 1, and statistics in Supplementary Data File 2. Colors represent the strength of mAbs resistance: strong (mFRN > 100); moderate (mFRN = 10–100); mild (mFRN = 3–10); no resistance (mFRN = 1–3); increased sensitivity (mFRN < 1). Bam + Ete, bamlanivimab + etesevimab; REGEN-COV (Cas + Imd), casirivimab + imdevimab; Evusheld (Tix + Cil), cilgavimab + tixagevimab; NA, no neutralization data reported for the antibody-variant pair at the time of writing.

Numerous mutations continuously congregated within the RBD region of SARS-CoV-2. Some emerging strains with specific combination of RBD mutations originated from “variant soup” (Callaway, 2022) could partially or completely impair mAb recognition while still retaining or even enhancing viral infectivity. With the enhanced fitness, some SARS-CoV-2 variants obtained great potential to rapidly spread among huge populations and became dominant strains (Wang et al., 2021). Several variants of concern (VOCs), such as Alpha (Supasa et al., 2021), Beta (Zhou et al., 2021), Gamma (Dejnirattisai et al., 2021), Delta (Liu et al., 2021), Omicron (Dejnirattisai et al., 2022) and its numerous subvariants, have been carefully monitored (Fig. 1A). Some VOCs have caused regional pandemic, such as Beta in South Africa and Gamma in Brazil, respectively; while others, such as Omicron and its subvariants, turned into a huge worldwide pandemic with continuous waves of infection cases (Dejnirattisai et al., 2022; Fan et al., 2022; Hoffmann et al., 2021; Wilkinson et al., 2021). Various Omicron sublineages, including BA.1, BA.2, BA.4/5 (identical point mutations in RBD between BA.4 and BA.5), BA.2.75, and BQ variants have dominantly replaced other VOCs, resulting in a multiwave pandemic dynamics (Jian et al., 2022; Qu et al., 2023a; Sheward et al., 2022; Shu and McCauley, 2017). Moreover, frequent recombination events elicited new recombinant sublineages. For example, XBB variant was likely originated through a recombination of two Omicron subvariants, BJ.1 and BM.1.1.1 (Tamura et al., 2023a) (Fig. 1A). Nowadays, SARS-CoV-2 is still mutating, making it hard to predict the direction of S protein evolution (Qu et al., 2023c; Scarpa et al., 2023; Tamura et al., 2023b; Wang et al., 2023b).

Due to its significance during viral entry, the RBD not only becomes a promising target for vaccine design and monoclonal antibody (mAb) development, but also encounters powerful selective pressure (Chen et al., 2023; Cox et al., 2023; Tregoning et al., 2021; Xu et al., 2022). Amino acid substitution at RBD may influence the chemical and physical properties of S protein, finally altering its functional characteristics, including protein expression (Starr et al., 2022a), protein stability (Starr et al., 2020) and protein-protein interactome (Mannar et al., 2022; Motozono et al., 2021; Shuai et al., 2022; Yin et al., 2022; Zhang et al., 2020, 2021a). Particularly, ACE2-S binding affinity and neutralization resistance are the two major outcome dimensions, indicating the infection efficiency and immune evasion capacity, respectively, for each emerging SARS-CoV-2 variant during evolution (Huo et al., 2023; Ma et al., 2023; Zhao et al., 2023).

In this review, we discuss the effect of VOC mutations on both ACE2 affinity and antibody resistance, aiming to reveal how RBD point mutations coordinate and achieve a balance between these two viral characteristics. We review how specific RBD mutations enable further emergence of escape mutations by compensating their deleterious effect for ACE2 affinity (e.g., N501Y), and also restrict overall sequence variation (e.g., almost all Omicron sublineages contain Q498R-N501Y double mutation).

## Methods to characterize various RBD mutations

To measure the ACE2 binding affinity for S proteins of distinct VOCs *in vitro*, surface plasmon resonance (SPR) (Piliarik et al., 2009) and biolayer interferometry (BLI) (Kumaraswamy and Tobias, 2015) techniques have been wildly used. Both of these techniques utilize sensitive optical biosensors to capture minor signals generated by the association and disassociation of target molecules in real time and calculate the affinity constant (*K*_D_) to represent the S-ACE2 binding affinity (Han et al., 2022; Li et al., 2022; McCallum et al., 2022; Zhang et al., 2021a). Therefore, by comparing the *K*_D_ value of a mutated S protein with that of a wildtype S protein under the same experimental condition, the effect of mutations on ACE2 binding affinity could be precisely measured and presented as the average change in logarithmic negative *K*_D_ [Δ−log(*K*_D_)].

However, it is worth noting that the native interaction between trimeric S proteins and dimeric ACE2 receptors *in vivo*, such as the RBD open/closed conformations, could not be fully and precisely represented by the SPR/BLI assays, which usually utilize only recombinant RBD protein and monomeric ACE2 (Walls et al., 2020; Yan et al., 2020). Besides, even utilizing S and ACE2 proteins under the same form, the absolute *K*_D_ value also varied among different assays or studies (Dejnirattisai et al., 2021; Li et al., 2022; Zhang et al., 2021a).

To evaluate the antibody evasion capacity, the *in vitro* neutralization assays are usually performed in the presence of different concentrations of mAbs (Muruato et al., 2020; Nie et al., 2020; Zhou et al., 2022). Based on the neutralization curve, an IC_50_ value (concentration of a mAb to neutralize 50% virus in the neutralization assay) could be calculated to show the absolute neutralization potency for each mAb-variant pair (Cox et al., 2023). Moreover, by comparing the IC_50_ value of a mAb against a variant pseudovirus with the IC_50_ value for the wildtype pseudovirus as a reference, the fold reduction in neutralization (FRN) could be calculated (Cox et al., 2023). Thus, the geometric mean of IC_50_ and FRN (mFRN) values for a given mAb-variant pair reflects the absolute neutralizing activity and the relative reduction level of neutralization.

Although SPR/BLI analysis and *in vitro* neutralization assays are performed to reveal the virus characteristics for different SARS-CoV-2 variants, there are limitations of these assessments that the experiment scale is too small. The fully mutated S proteins only reveal the combined effect of single mutations but could not elaborate the extent of the effect of each individual RBD mutations or their various combinations on viral characteristics. Thus, it would be very meaningful and crucial to dissect out the effect of each individual mutation or their various combinations.

To address this need, deep mutation scanning (DMS) approaches, which conventionally integrate a yeast-surface display platform using a mutagenesis library of spike RBD with the fluorescence-activated cell sorting and deep sequencing techniques, have been developed (Cao et al., 2023; Frank et al., 2022; Greaney et al., 2021c; Starr et al., 2020; Taylor and Starr, 2023). Very recently, a novel DMS platform utilizing pseudovirus mutagenesis library instead of yeast has also been established to examine the immune evasion capacity of full spike (Dadonaite et al., 2023). Various strategies for library construction facilitate the high-throughput investigation to reveal the effect of point or combined mutations, even including all possible RBD substitutions (Greaney et al., 2021c; Moulana et al., 2022; Starr et al., 2022a). Moreover, DMS techniques exhibit reliable reproductivity and consistent output results as the same as conventional affinity and neutralization assays (Greaney et al., 2021b; Tan et al., 2023). Therefore, the established DMS systems enable high-throughput screening of functional alterations induced by S protein mutations and could greatly contribute to the understanding of the evolving SARS-CoV-2 mutants (Cao et al., 2022b).

Together, SPR/BLI analysis using the recombinant S proteins could be performed to measure the S-ACE2 binding affinity [*K*_D_ value and Δ−log(*K*_D_)], while the pseudoviruses-based *in vitro* neutralization assays could be carried out to evaluate the antibody neutralization potency and breadth (IC_50_ and mFRN values). Combined with DMS techniques, the impacts of single RBD mutations (in wildtype background) on ACE2 affinity and mAbs resistance could be evaluated in a high-throughput, more precise, and less-biased way. With all these data and tools, we could characterize and compare the survival advantages for each different SARS-CoV-2 variant (Fig. 1B and 1C).

## ACE2 affinity and antibody evasion capacity of distinct VOCs

We summed up 36 studies (up to 10 June 2023) about SARS-CoV-2 VOCs and other newly emerging variants in regard to their ACE2 binding affinity (Supplementary Data File 1). We further summed up 132 studies (118 studies concluded in one review article (Cox et al., 2023) and additional 14 recent individual studies) related to the neutralizing activity of RBD-targeting mAbs (Supplementary Data File 1).

In this review, we mainly discuss 11 mAbs, including casirivimab, etesevimab and tixagevimab in Class-1 RBD epitope; bamlanivimab and cilgavimab in Class-2 RBD epitope; bebtelovimab, imdevimab and sotrovimab in Class-3 RBD epitope; cocktail of bamlanivimab and etesevimab; Evusheld (cocktail of tixagevimab and cilgavimab); and REGEN-COV (cocktail of casirivimab and imdevimab) (Fig. 1C). Of note, there are other groups of mAbs characterized by their distinct RBD epitopes. For instance, seven RBD epitopes in (Hastie et al., 2021) and 12 distinct RBD epitopes in (Cao et al., 2022c). However, the chosen 11 mAbs to be discussed in this review have been authorized by the U.S. Food and Drug Administration (FDA) for the emergency use authorization (EUA) as therapeutic drugs and they have been very well studied and characterized, including their neutralizing activity against various SARS-CoV-2 variants and their structural details of antibody–antigen interfaces.

In SARS-CoV-2 VOC Alpha, its S protein with only one RBD mutation N501Y, showed significant enhancement in ACE2 affinity relative to the wildtype S protein [Δ−log(*K*_D_) = 0.64, 4.3-fold enhancement] (Fig. 1B). This observation explained the enhanced SARS-CoV-2 infection and transmission induced by the N501Y substitution(Liu et al., 2022). Alpha variant showed very low level of antibody evasion, with only few mAbs losing neutralizing activity against Alpha. For example, only etesevimab, which directly contacts the RBD mutation site in the S protein of Alpha, showed a mild reduction in neutralizing Alpha (mFRN = 10.2; IC_50_ = 150 ng/mL) (Fig. 1C).

In SARS-CoV-2 VOC Beta and Gamma, their S proteins both possess mutations at the K417, E484, and N501 amino acid positions in the RBD, thus exhibiting similar mAbs evasion pattern. Specifically, among the 11 FDA-authorized EUA antibodies, the same five mAbs (casirivimab, etesevimab, tixagevimab, bamlanivimab, and bamlanivimab/etesevimab cocktail) showed reduced or abrogated neutralizing activity against both Beta and Gamma (Fig. 1C). However, despite their identical mutation positions in the RBD, Gamma [Δ−log(*K*_D_) = 0.68] presented a 1.6-fold higher ACE2 affinity than Beta variant [Δ−log(*K*_D_) = 0.46] (Fig. 1B). This observation was probably due to their different amino acid substitution strategy for lysine at position 417 (K417). Beta variant chose a lysine-to-asparagine (K417N) substitution here, while Gamma used threonine (K417T), which has less harmful effect on ACE2 binding (Starr et al., 2022b) (Fig. 1A).

In SARS-CoV-2 VOC Delta, S protein exhibited comparable or slightly enhanced affinity to ACE2 compared with the wildtype S protein [Δ−log(*K*_D_) = 0.04, indicating 1.1-fold stronger binding affinity]. This observation suggested that the combination of mutations, L452R and T478K in Delta RBD, exerted limited influence on receptor engagement. As for antibody evasion, Delta remained sensitive to most mAbs. Among the 11 FDA-approved mAbs, 9 mAbs maintained neutralizing activity with their mFRN < 3; while only 2 mAbs bamlanivimab (mFRN = 477.58; IC_50_ = 3,756 ng/mL) and cilgavimab (mFRN = 3; IC_50_ = 17.86 ng/mL) lost the neutralizing activity (Fig. 1C).

Of note, a higher mFRN value only indicates a greater reduction of neutralizing efficiency for the given mAb-variant pair relative to the wildtype control, and should not be interpreted as an absolute low level of neutralizing activity. For instance, two mAbs, cilgavimab (mFRN = 3) and imdevimab (mFRN = 1.98), had higher mFRN values against Delta than mAb sotrovimab (mFRN = 1.3), but both cilgavimab (IC_50_ = 17.86 ng/mL) and imdevimab (IC_50_ = 19.71 ng/mL) exhibited much more potent neutralizing activity against Delta than sotrovimab (IC_50_ = 106.68 ng/mL) (Fig. 1C). Therefore, IC_50_ and mFRN values should be combined to analyze mAb neutralizing potency and to evaluate the evasion capacity of different variants.

For SARS-CoV-2 VOC Omicron and its sublineages, their S proteins with numerous RBD mutations exhibited striking antibody evasion characteristics. Among the 11 FDA-authorized EUA mAbs, 8 products including 2 mAb cocktails (cocktail of bamlanivimab and etesevimab; REGEN-COV cocktail of casirivimab and imdevimab) showed a remarkable reduction of neutralization (mFRN > 100) against BA.1; while the rest 3 products (bebtelovimab, sotrovimab and Evusheld cocktail) retained, partially if not fully, their anti-BA.1 neutralization capacity (mFRN = 1.15, 3.17, and 69.65; IC_50_ = 2.77, 281.09, and 261.92 ng/mL, respectively) (Fig. 1C). For variant BA.2, it could still be effectively neutralized by cilgavimab, bebtelovimab, sotrovimab, and Evusheld cocktail (mFRN = 2.53, 1.00, 21.41, and 7.84, respectively), but had been found to completely evade neutralization by tixagevimab (mFRN > 1000; IC_50_ = 4,057.43 ng/mL). Despite tixagevimab was evaded by BA.2, Evusheld cocktail (cilgavimab plus tixagevimab) retained its neutralizing potency due to the fact that cilgavimab remained functional, emphasizing the significance of combining mAbs recognizing different epitopes into cocktail. This multivalent antibody strategy could reduce antibody evasion and extend the breadth of mAb therapy. BA.4, BA.5, and BA.2.75 subvariants further evolved to extend antibody evasion. Consequently, among the 11 FDA-authorized EUA mAbs, only bebtelovimab retained the neutralization capacity (mFRN < 10; IC_50_ = 2.80 ng/mL for BA.4/5; IC_50_ = 25.19 ng/mL for BA.2.75) against these lately emerging variants (Fig. 1C).

Interestingly, along with the antibody evasion, BA.1, BA.2, BA.4/5 and BA.2.75 showed successive improvement for their S-ACE2 binding affinity [Δ−log(*K*_D_) = 0.16, 0.42, 0.63 and 1.01, respectively] (Fig. 1B). Especially, the S protein of BA.2.75, exhibiting over 10-fold stronger ACE2 affinity than the wildtype S protein, reached the highest binding activity among all variants discussed here. Accordingly, when compared with other circulating SARS-CoV-2 strains (such as BA.4/5), BA.2.75 displayed obvious growth advantage in India after it was initially detected (Cao et al., 2022a).

Recently emerged BQ and XBB subvariants completely evaded almost all clinically available mAbs (mFRN > 1000) except for sotrovimab (Addetia et al., 2023; Driouich et al., 2023). Sotrovimab, derived from the parental mAb S309 and bearing the LS modification that extends antibody half-life, partially retained the capacity to neutralize BQ.1.1 and XBB.1 (mFRN = 83.58 and 12.93; IC_50_ = 4,772.52 and 590.44 ng/mL, respectively) (Addetia et al., 2023; Bruel et al., 2023; He et al., 2023; Planas et al., 2023). However, the receptor binding affinities for BQ.1.1 [Δ−log(*K*_D_) = 0.76] and XBB.1 [Δ−log(*K*_D_) = 0.16] were not as high as that of BA.2.75 [Δ−log(*K*_D_) = 1.01]. These findings suggested that some of the additional RBD mutations like K444T in BQ.1.1 and V445P in XBB.1 exert negative influence on ACE2 binding but promote antibody evasion (Qu et al., 2023a; Wang et al., 2023b).

XBB.1.5 subvariant presented dominating global prevalence in early 2023. Preliminary data showed that, compared with its parental strain XBB.1, XBB.1.5 has achieved comparable mAbs resistance and much stronger ACE2 affinity [Δ−log(*K*_D_) = 0.84]. However, many virological characteristics of XBB.1.5 has not been understood very well, and more detailed studies are still required to comprehensively assess its ACE2 affinity and antibody evasion level. For example, since the F486P mutation constitutes the only specific alteration in XBB.1.5 RBD relative to XBB.1, the mechanism underlying the F486P-induced enhancement of ACE2 receptor binding needs to be further revealed structurally (Uriu et al., 2023; Yue et al., 2023).

Together, on one hand, all VOCs discussed here strengthen, to varying degrees, their S-ACE2 binding affinity relative to the prototype, while on the other hand, the general trend of SARS-CoV-2 viral evolution to efficiently evade neutralization by antibodies elicited upon infection or vaccination is expected to persist. Accordingly, the assessment of both receptor affinity and antibody resistance capacity is crucial for the comprehensive analysis of the newly emerged SARS-CoV-2 variants. In the future, the SARS-CoV-2 virus will keep mutating, leading to a shortage of antibodies for clinical treatment. Meanwhile, it is very possible that a novel SARS-CoV-2 variant with superior ability to recognize ACE2 and evade antibody neutralization would trigger a new round of pandemic. Therefore, it is crucial to maintain vigilant surveillance of viral evolution and keep developing broad neutralizing antibodies targeting conserved epitopes and neutralizing all known variants.

## Key mutations for antibody resistance

**Figure 2.**
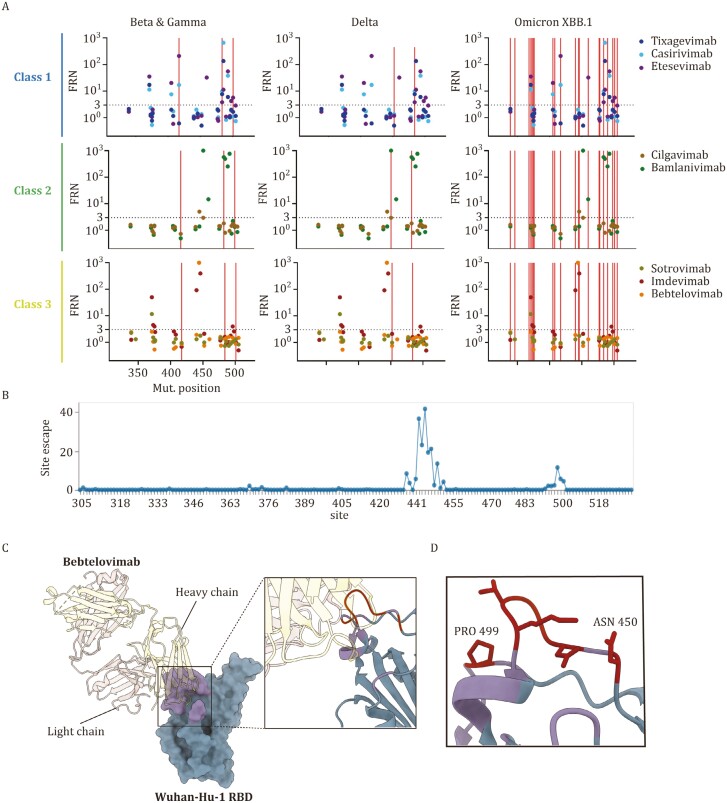
**Epitope mutations drive resistance to antibody neutralization.** (A) Geometric mean fold reduction in neutralization (mFRN) data for each class of monoclonal antibody (mAb). The mFRN values (y-axis) was determined using the wildtype pseudoviruses containing only one single mutation in the RBD of S protein (positions 305–534, x-axis) (Shang et al., 2020), with the wildtype pseudovirus as the reference control. Each dot represents the mFRN value of one mutation-mAb pair (mutation sites refer to Fig. 1A). The colors of dots represent the corresponding testing mAbs. Horizonal dashed line shows the mFRN = 3 threshold. Mutation sites of representative VOCs Beta, Gamma, Delta and Omicron XBB.1 (see Fig. 1A) are indicated by vertical red lines. The full dataset of mFRN values for each mAb in the presence of single RBD mutations are summarized in the Supplementary Data File 3. (B) Total escape scores of bebtelovimab (LY-CoV1404) determined by a full spike deep mutational scanning system at each site in the BA.1 RBD. A detailed explanation of escape score could be found in the reference (Yu et al., 2022). An interactive data set is available at Github. (C) X-ray crystal structure of the bebtelovimab Fab bound to the S protein RBD (PDB 7MMO). The rectangular region indicates the Wuhan-Hu-1 RBD epitope recognized by bebtelovimab and is showed as ribbons in the zoomed view. The key escape sites, such as N450 and P499, correspond to the region (site 444-450 and 499) with escape score > 10 (see Fig. 2B). (D) Key escape sites in (C) are showed as atoms.

Despite their distinct RBD mutation profiles, the newly emerging Omicron variants undergo convergent evolution that accumulate mutations at several hotspots (Addetia et al., 2023; Bouhaddou et al., 2023; Cao et al., 2023; Focosi et al., 2023; Martin et al., 2021; Qu et al., 2023b). This signature of SARS-CoV-2 evolution is mainly attributed to the pressure of humoral immune response, thus many point mutations induce viral resistance to mAbs (Chen et al., 2021; Greaney et al., 2021a, 2021b; McCallum et al., 2022; Planas et al., 2023; Tuekprakhon et al., 2022). To better analyze the impact of individual RBD point mutations in antibody evasion, we collected the published FRN values for a series of single mutants in the wildtype background and overlaid them into plots containing VOC mutation positions (Fig. 2A).

SARS-CoV-2 VOCs Beta (K417N, E484K and N501Y in RBD) and Gamma (K417T, E484K and N501Y in RBD) possess RBD mutations at the same positions. Class 1 mAbs, casirivimab and etesevimab, showed a reduction of neutralization in the presence of K417N (mFRN = 17 for casirivimab and 210 for etesevimab, respectively) or E484K (mFRN = 15 for casirivimab and 3 for etesevimab, respectively) individually, thus exhibited a combined impaired neutralization against Beta (mFRN = 102 and 414, respectively). For Gamma, RBD mutation K417T also rendered resistance to these two mAbs (mFRN = 7 and 32, respectively), thus similar effects of antibody evasion were also observed for Gamma (Fig. 2A). Therefore, Beta and Gamma, with RBD mutations at the same positions, showed a similar mAb evasion pattern (Fig. 1C).

Besides Class 1 mAbs, casirivimab and etesevimab, E484K mutation also significantly contributed to resistance against Class 2 mAb, bamlanivimab (mFRN = 751). Mechanistically, structural investigations have shown that E484K significantly alters the electrostatic complementarity for antibody binding (Andreano et al., 2021). Thus, bamlanivimab showed a reduced neutralization efficacy against both Beta and Gamma.

Correspondingly, one mAb is expected to maintain their neutralization efficacy when the target variant possesses no individual mutation to resist the neutralizing activity of this mAb. For example, in Delta variant, none of its RBD mutations showed significant resistance to sotrovimab, imdevimab, and bebtelovimab, making all these three Class 3 mAbs still neutralizing against Delta (mFRN = 1.3, 2, and 1.4, respectively).

Similar principles also apply to mAb cocktails. For instance, the mutations S371F and K417N evaded neutralization by casirivimab (mFRN = 11 and 17, respectively), while mutations S371F, N440K and G446S evade imdevimab (mFRN = 50, 92, and 394, respectively). Consequently, the Omicron subvariant XBB.1, containing S371F, K417N, N440K, and G446S, completely escaped the REGEN-COV cocktail (casirivimab + imdevimab, mFRN > 1000) (Fig. 1C). These data suggest that distinct groups of escape mutations escape the neutralization of each individual mAb within the cocktail, and the combination of all mutations lead to the complete escape against mAb cocktail.

Mutations that confer the highest resistance to a mAb were observed to predominately cluster at the epitope positions where the mAb binds in RBD (Cox et al., 2023; Greaney et al., 2021b). A novel DMS platform utilizing pseudovirus mutagenesis library have revealed the key escape mutations for bebtelovimab, a potent Class 3 mAb (Alcantara et al., 2023; Dadonaite et al., 2023; Starr et al., 2022b; Westendorf et al., 2022). Several potential escape mutations, located within the antibody epitope, site 444–450 and 499, exerted striking antibody evasion against bebtelovimab (Dadonaite et al., 2023) (Fig. 2B, 2C, and 2D). *In vitro* pseudovirus neutralization assays verified that a single K444T mutation located at the interface was sufficient to fully evade bebtelovimab (mFRN > 1000) (Dadonaite et al., 2023).

However, some escape mutations are outside of the antibody-antigen interface. For instance, S371F substitution, located outside of the binding epitope of these mAbs, exhibited moderate evasion of sotrovimab and imdevimab (mFRN = 12 and 50, respectively). Structural studies provided an explanation that this mutation results in the rearrangement of RBD helix comprising residues 364 to 372, adopting a distinct conformation (Park et al., 2022). Similarly, substitutions S373P and S375F, outside of the interface of imdevimab, also induced conformational changes to alter antigenic characteristics, so the recognition by imdevimab is hampered (mFRN = 4.4 and 4, respectively) (Cui et al., 2022). Thus, besides the interface mutations, the non-interface mutations might affect the global spike conformation to disrupt the antibody neutralization.

Collectively, SARS-CoV-2 variants could exhibit resistance to a specific mAb only when they have acquired certain point escape mutations, and these point mutations individually contribute to the antibody evasion, irrelevant of their S protein backbone. Therefore, it is concluded that a certain point mutation has comparable antibody evasion capacity regardless of their variant background. This phenomenon contradicts with the notion of “epistasis” observed for the mutations affecting ACE2 affinity, which will be discussed in the following section in this review.

## Epistatic mutations for rescuing ACE2 affinity

**Figure 3.**
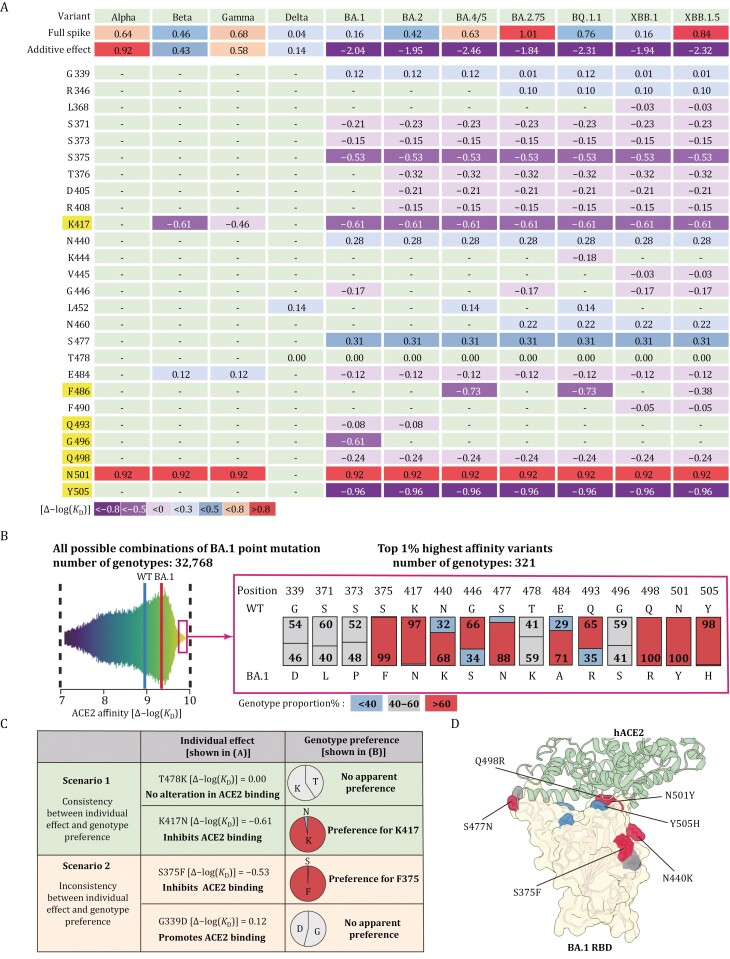
**Potent epistatic mutations reverse the deleterious summed effect on ACE2 affinity.** (A) Heat map depicting relative enhancement in ACE2 affinity for variants of concern (VOCs) (top row) and individual mutations (other rows). Each column shows data for each variant, including Alpha, Beta, Gamma, Delta, BA.1, BA.2, BA.4/5, BA.2.75, BQ.1.1, XBB.1, and XBB.1.5. The data in each row are the calculated logarithmic negative *K*_D_ [Δ−log(*K*_D_)] value, to present the effect of mutations on ACE2 binding affinity. These values are calculated by comparing the *K*_D_ value of a mutated S protein with that of a wildtype S protein under the same experimental condition. The effect for each constituent VOC mutation individually on a wild type background, together with their summed effect is shown. At the left-most column of the table, the original amino acids of the S protein and their corresponding positions are shown; and the amino acids (K417, F486, Q493, G496, Q498, N501, and Y505) directly contacting ACE2 are highlighted. Different colors represent the changed levels on ACE2 affinity: strong enhancement [Δ−log(*K*_D_) > 0.8]; moderate enhancement [Δ−log(*K*_D_) = 0.5–0.8]; mild enhancement [Δ−log(*K*_D_) = 0.3–0.5]; slightly enhancement [Δ−log(*K*_D_) < 0.3]; slightly decreased affinity [Δ−log(*K*_D_) = −0.5–0]; moderate decreased affinity [Δ−log(*K*_D_) = −0.5–−0.8]; strongly decreased affinity [Δ−log(*K*_D_) < −0.8]. “−” indicates no mutation at this position for the corresponding variants/column.(B) Systematic analysis of ACE2 binding affinity for RBD proteins containing all possible combinations of 15 mutations in the RBD of BA.1 variant. Left: distribution of ACE2 binding affinity using all possible mutational intermediates of BA.1 (N = 2^15^ = 32,768 RBD genotypes tested). Binding affinity is shown as −log(*K*_D_). The vertical lines indicate the −log(*K*_D_) for wildtype Wuhan-Hu-1 strain and Omicron BA.1 variant, respectively. An interactive data browser is available at Github. Right: among the top 1% intermediates with a superior ACE2 affinity (a total of 321 genotypes), relative proportions (%) of each amino acid are shown at all 15 mutation sites. On these RBD positions, the amino acids for BA.1 variant and the corresponding amino acid in wildtype Wuhan-Hu-1 strain are also shown. Colors depict the genotypes preference at each position: preferred (genotype proportion > 60%); no apparent preference (genotype proportion = 40%–60%); unappreciated (genotype proportion < 40%). (C) Two scenarios when comparing the effect of individual BA.1 constituent mutations in the wildtype backbone [Δ−log(*K*_D_) values from Fig. 3A] with their proportions among the top 1% variants with highest ACE2 affinity (genotype proportions from Fig. 3B). (D) Co-crystal structure of Omicron BA.1 RBD and ACE2 receptor (PDB ID 7WPB). Mutated residues are shown, and their surfaces are colored as the corresponding residues in Fig. 3B.

The ACE2 affinity of S protein is of primary importance for SARS-CoV-2 infection, thus distinct VOC mutations work together to maintain the ACE2 affinity and even enhance it to varying degrees (Cao et al., 2022a; Chakraborty, 2022; Liu et al., 2022; Ma et al., 2023; McCallum et al., 2022; Ozono et al., 2021). We compared Δ−log(*K*_D_) values of VOCs and examined the summed effects of each individual constituent mutations in the wildtype backbone (Fig. 3A).

The DMS profile showed that the N501Y substitution (the only mutation harbored in Alpha RBD) conferred an 8.3-fold enhancement in ACE2 affinity [Δ−log(*K*_D_) = 0.92] (Fig. 3A). This was consistent with the result of individual SPR assay (Barton et al., 2021). However, the actual ACE2 affinity with Alpha S protein was only 4.4-fold stronger than that of the wildtype S protein [Δ−log(*K*_D_) = 0.64]. This inconsistency could probably be explained by the mutations outside of the RBD, which inhibit ACE2 binding capacity and lead to the overall decrease of ACE2 affinity.

Despite this, the cumulative impact of RBD mutations in the Beta, Gamma, and Delta variants [Δ−log(*K*_D_) = 0.43, 0.58, and 0.14, respectively] aligned closely with the ACE2 affinities observed in their full-length complete spike proteins [Δ−log(*K*_D_) = 0.46, 0.68, and 0.04, respectively] (Fig. 3A). This observation explains the stronger ACE2 affinity of Gamma compared with Beta and Delta. Since the only difference of RBD sequence between Beta and Gamma is the K417 substitution (K417N harbored by Beta while K417T within Gamma), K417N in Beta led to a greater reduction in ACE2 affinity than K417T [Δ−log(*K*_D_) = −0.61 for K417N and −0.46 for K417T, respectively].

Together, among these RBD mutations in Alpha, Beta, Gamma, and Delta, N501Y exhibited the most significant enhancement in ACE2 affinity, while K417N substitution led to the greatest reduction (Barton et al., 2021; Han et al., 2022; Mannar et al., 2021). For these SARS-CoV-2 variants, the mutations independently influence ACE2 affinity, and the overall ACE2 binding affinity is the combined effect of all individual mutations.

Nevertheless, the situation for Omicron and its subvariants with multiple RBD mutations seemed more complex. As revealed by DMS analysis, most of these mutations alone inhibited ACE2 binding within wildtype background. For instance, S375F, G496S, Y505H, and aforementioned K417N substitutions, individually in a wildtype backbone, have been found to significantly impair the ACE2 binding [Δ−log(*K*_D_) = −0.53, −0.61, −0.96, and −0.61, respectively] (Cao et al., 2022b; Greaney et al., 2021b, 2022). It was therefore speculated that the 15 RBD mutations in Omicron variant BA.1 cumulatively cause greater than 100-fold reduction in ACE2 binding [Δ−log(*K*_D_) = −2.04] (Fig. 3A).

However, surprisingly, the fully mutated spike proteins of Omicron subvariants managed to maintain or even strengthen their ACE2 affinity compared with the wildtype control, completely deviating from the summed effect of their constituent mutations (Fig. 3A). This obvious and typical “epistasis” phenomenon indicates the complex interaction among Omicron RBD mutations (Starr and Thornton, 2016). In another word, the actual effect of a single point mutation on ACE2 binding depends on the specific amino acid sequence of the whole spike protein.

Taking N501Y mutation as an example, it exerted an enhancement effect on ACE2 affinity for various SARS-CoV-2 variants but severely impaired ACE2 binding in SARS-CoV-1 or other sarbecoviruses (Starr et al., 2022c). Therefore, N501Y, as an RBD mutation, exhibited a distinct effect on ACE2 binding in different S protein background.

On the other hand, certain mutations could also affect ACE2 affinity very differently in an N501Y-positive background versus an N501Y-negative background. In a recent study, DMS analysis studied the effects of a series of single mutations in different VOC backgrounds and revealed that the epistatic effects on ACE2 binding are mainly attributable to the N501Y substitution (Starr et al., 2022a). Specifically, in N501Y-containing Alpha (N501Y only in RBD) and Beta (K417N, E484K, and N501Y in RBD) background, several RBD mutations, such as Q498R, one of RBD mutations in Omicron, showed significantly enhanced ACE2 binding than that in N501Y-absent wildtype background, suggesting that these RBD sites exhibited positive epistatic shifts in the presence of N501Y (Starr et al., 2022a). Conversely, RBD mutations in the Delta (L452R and T478K in RBD) background barely altered the ACE2-binding affinity compared with those in wildtype RBD background, suggesting that no such epistatic shift for RBD mutations was observed in the absence of the N501Y substitution (Starr et al., 2022a). Since most of the popular Omicron sublineages possess the N501Y mutation, its strong epistatic effect might reverse the negative effect on ACE2 binding for other RBD mutations and be of great significance in maintaining the overall ACE2 affinity (Fig. 3A).

Systematical evaluation of the effect of point mutations on ACE2 affinity in different variant contexts even before their actual appearance successfully unraveled the epistasis phenomena. However, as new point mutations emerge and accumulate in newly emerged SARS-CoV-2 variants, it becomes crucial to understand the effect of not only single but also combinational mutations.

A recent study constructed a mutagenesis library containing all possible combinations of 15 mutations in the RBD of BA.1 variant (a total of 2^15^ = 32,768 genotypes of variants) and measured their ACE2 binding affinity to capture the epistatic interactions among these mutations (Moulana et al., 2022). Although all these mutational variants exhibited ACE2 binding, only a small proportion showed an enhanced ACE2 binding affinity compared with the wildtype RBD (Fig. 3B, left). Interestingly, among the 321 (the top 1%) genotypes with extraordinarily strong ACE2 binding capacity, the relative mutation composition (%) at the 15 BA.1 mutation sites are radically different (Fig. 3B, right). For example, all 321 sequences had Q498R and N501Y, indicating the top 1% variants with a superior ACE2 affinity exhibited a strong preference of Q498R-N501Y double mutation (Moulana et al., 2022) (Fig. 3B). This observation is consistent with a 387-fold enhancement of ACE2 affinity for this double mutation-containing RBD (Starr et al., 2022a).

We further analyzed these mutation biases (ratio values in Fig. 3B) in combination with the effect of these individual mutations in the wildtype background (Fig. 3B), and found two scenarios (Fig. 3C). (1) It is consistent between the effect of the individual mutation on ACE2 affinity and the selection preference observed among the top 1% ACE2-binding variants. For example, a K478 substitution showed no alteration in ACE2 binding when individually introduced into wildtype backbone [Δ−log(*K*_D_) = 0]. Consistently, no apparent preference was observed between T478 and K478 among the 321 genotypes of the top 1% ACE2-binding variants. Another example is K417. Among the 321 genotypes of the top 1% ACE2-binding variants, K417 accounted for 97%. Correspondingly, compared with N417 substitution in a wildtype background [Δ−log(*K*_D_) = −0.61], the K417 showed stronger ACE2 binding. (2) An inconsistency was observed between the individual effect and selection preference. For example, a F375 substitution largely dampened ACE2 binding when individually introduced into wildtype backbone [Δ−log(*K*_D_) = −0.53]. However, among the 321 genotypes of the top 1% ACE2-binding variants, F375 accounted for 99% in the presence of Q498R-N501Y double mutations, suggesting the beneficial effect of F375 on ACE2 affinity. Another example is D339, which boosted the ACE2 binding in the wildtype backbone; but showed no preference compared with G339 among the 321 top 1% variants.

Together, these results suggest that the reduction effect on ACE2 binding could be compensated or reversed by the presence of Q498R-N501Y (scenario 2 in Fig. 3C), emphasizing the positive epistasis effect induced by Q498R-N501Y double mutations. However, several mutations showed a similar impact as those seen in the wildtype (scenario 1 in Fig. 3C).

Q498R and N501Y collaborate to exert potent epistasis effect on maintaining ACE2 affinity, and they also confer resistance to etesevimab (mFRN = 4 and 6, respectively), suggesting that some mutations could simultaneously contribute to ACE2 binding and antibody evasion.

Apparently, combinatorial assembly study to assess the ACE2 binding affinity for all possible mutation combinations is a valuable tool to discover the variants with the utmost ACE2 affinity and to pinpoint mutations inducing positive epistatic effect (Fig. 3D).

It is worth noting that certain BA.1 mutations, such as K417N (for antibody evasion) and Y505H (for mouse ACE2 adaption), have no enhancement effect on ACE2 affinity, or even harm the ACE2-S binding affinity. However, these mutations are consistently preserved in the recently emerged SARS-CoV-2 variants (Yuan et al., 2021; Zhang et al., 2022), suggesting that to enhance the ACE2 affinity is not the only direction for SARS-CoV-2 evolution. Considering many mutations formed for antibody evasion or host adaptation, the appearance of positive epistatic mutations could effectively mitigate the harmful effect of these mutations on ACE2 affinity; and as a result, maintenance of sufficient ACE2 binding affinity provides more potential for various S protein mutations to confer more significant survival advantages (Javanmardi et al., 2022; Zhang et al., 2022).

## Conclusions and perspectives

**Figure 4.**
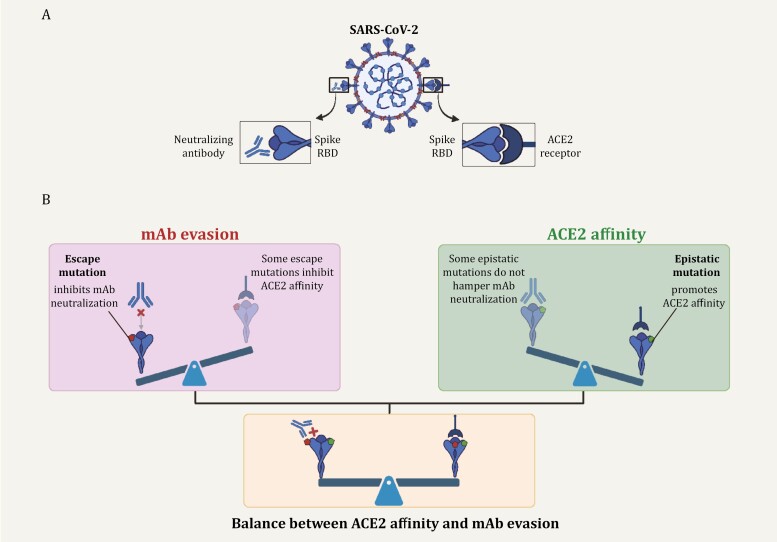
**Maintaining the balance between ACE2 affinity and mAb evasion for the viral fitness.** (A) To inhibit infection, the neutralizing antibody competitively binds to SARS-CoV-2 RBD, while the interaction between the viral S protein and the host receptor ACE2 is abolished. Created with Biorender. (B) Upper left: In the presence of certain escape mutation, a neutralizing antibody might lose its neutralizing capacity. However, some escape mutations could also decrease the ACE2-S binding affinity, reducing the viral infectivity and fitness. Upper right: some RBD mutations might exert epistatic effects to enhance ACE2 binding. Bottom: For each SARS-CoV-2 variant, a delicate balance needs to be kept between its immune evasion capacity (achieved through escape mutations) and sufficient ACE2 binding affinity (maintained by epistatic mutations). Created with Biorender.

Random sequence alterations introduce amino acid mutations on the spike protein of SARS-CoV-2. Combined with selective pressure from host humoral and cellular immune responses, the SARS-CoV-2 virus continues to evolve and novel variants with specific mutation profiles keep merging. A series of RBD mutations display a significant antibody evasion capacity, and some of these mutations meanwhile dramatically weaken the ACE2 binding activity (Fig. 4). However, for the full-length spike of various variants, this weakened effect is counterbalanced by the epistatic effect of specific mutations, such as Q498R-N501Y double mutation (Fig. 4B). This highlights the significant role of epistasis in mitigating the deleterious effect against ACE2 affinity and maintaining sufficient ACE2-binding capacity for viral fitness.

Moreover, phylogenic studies have revealed that new substitutions more often occurred in sequence backgrounds in which they would exert more favorable effect on ACE2 affinity (Starr et al., 2022a). Interestingly, in a certain epistasis environment (e.g., the presence of epistatic mutations Q498R-N501Y), already existing mutations could be reversely mutated in the following evolutionary steps if they conduct deleterious ACE2 binding effect (Starr et al., 2022b). For example, R493 and S446 obtained by BA.1 were unfavorable compared with the unmutated wildtype version Q493 and G446 (see Fig. 3B). As expected, the R493Q reversion occurred in Omicron subvariants including BA.4/5, BA.2.75, BQ.1.1, and XBB (Xu et al., 2023) (Fig. 1A), while the S446G reversion in BA.2, BA.4/5, and BQ.1.1 (Fig. 1A).

Together, such phenomena emphasized that the presence of these epistatic mutations, due to their beneficial effect in maintaining ACE2 affinity, could simultaneously restrict the potential trajectories for the future viral evolution. The complicated interactions among mutations call for more intensive investigation so that we could better understand their combinational effects on overall viral characteristics.

As SARS-CoV-2 continues to evolve, it is worth noting that the overall pattern of epistasis may also drift due to the sequence variation. A recent DMS assay evaluated the epistatic shift between BA.2 RBD and XBB.1.5 RBD backbone and identified three sites (453, 455, and 456) with a significant functional alteration under an epistatic effect (Taylor and Starr, 2023). Specifically, the same F456L mutation decreased ACE2 affinity in BA.2 background but enhanced ACE2 binding in XBB.1.5 backbone. Combined with the fact that both the single F456L mutation and the “Flip” mutations (L455F-F456L double mutation) render extraordinary immune evasion capacity, it is not surprising to see these mutations frequently detected in recently emerged XBB sublineages, like EG.5 and HK.3 (Dyer, 2023; Faraone et al., 2023; Kaku et al., 2023; Qu et al., 2023c; Wang et al., 2023a; Zhang et al., 2023). In conclusion, the deleterious effect of escape mutations on ACE2 binding might be reversed by specific epistatic background, which again underscores the evolution trajectory to keep a balance between ACE2 affinity and immune evasion capacity (Fig. 4).

Furthermore, as data accumulates based on the standardized experiments, a more comprehensive assessment of viral characteristics could be accomplished by big data analysis. Recently, a generalizable modular framework named EVEscape has shown satisfying and promising results in predicting escape mutations (Rochman and Koonin, 2023). This framework combined a deep generative model trained on historical viral sequences with the structural and biophysical information to evaluate the escaping potential of specific mutations (Thadani et al., 2023). Similarly, high-quality datasets reflecting the ACE2 affinity as well as mAb evasion capacity of distinct spike sequences may also serve as vital resources in training deep learning model to more precisely predict the future trajectory of viral evolution.

Nevertheless, during the measurement of ACE2 affinity and antibody evasion capacity, one limitation should be taken into consideration. For *K*_D_ values, distinct assays (SPR, BLI, or DMS), recombinant proteins (full-length trimeric spike protein, spike ECD protein, or recombinant RBD protein), forms of ACE2 ligand (dimeric or monomeric) are all different parameters that could lead to data variability for the same variant (Barton et al., 2021; Javanmardi et al., 2021; Liu et al., 2022; Ramanathan et al., 2021). Similarly, despite the neutralization results in different systems tended to highly correlate (Cox et al., 2023; Riepler et al., 2020), various experimental conditions (pseudovirus concentration, host cell type, experimental output and so on) during neutralization assay could also generate considerable variability for FRN values (Chen et al., 2021; Schmidt et al., 2020; Wang et al., 2022). Although we tried to calibrate these values by utilizing the data of control virus strain (usually the Wuhan-Hu-1 or B.1 sequence), it is still needed to establish a standardized affinity measurement and neutralizing assay for a better comparison in the future (Knezevic et al., 2022).

In this review, we focus on RBD mutations as this region accounts for direct contact with ACE2 and is targeted by majority of therapeutic mAbs, and mainly discuss how the RBD mutations achieve the delicate balance between ACE2 affinity and antibody evasion (Fig. 4B). Besides, mutations within RBD could affect other spike characteristics, such as protein expression and spike stability (Gupta et al., 2021; Kemp et al., 2021; Kumar et al., 2021).

Moreover, mutations outside of RBD could also confer functional alteration and ultimately promote viral survival (Iketani et al., 2023; Kabinger et al., 2021; Liu et al., 2022; Saito et al., 2022). For instance, mutations in the amino-terminal domain (NTD) of spike protein might disturb antibody recognition (Chi et al., 2020; Ray et al., 2021), and mutations in the spike S2 domain, such as A942S, promote virus-host membrane fusion (Yang et al., 2022). D614G, as the most prevalent mutation of SARS-CoV-2, efficiently promotes S-ACE2 affinity, increases RBD “up” (open) state and enhances S1/S2 junction proteolysis, thereby contributing to SARS-CoV-2 fitness (Gobeil et al., 2021; Korber et al., 2020; Ozono et al., 2021; Plante et al., 2021). Obviously, a comprehensive assessment of ACE2 affinity and antibody evasion during viral evolution cannot be accomplished by analyzing RBD mutations alone, since the non-RBD mutations could also induce diverse effects. Therefore, it remains necessary to routinely monitor viral prevalence to timely identify dominant variants and to thoroughly assess the biological effects of the newly emerging point mutations and mutational combinations.

## Supplementary Material

pwae007_suppl_Supplementary_Files_1

pwae007_suppl_Supplementary_Files_2

pwae007_suppl_Supplementary_Files_3

## Data Availability

All data generated or analyzed during this study are included in this published article.
